# Effectiveness of COVID-19 Vaccination Mandates and Incentives in Europe

**DOI:** 10.3390/vaccines10101714

**Published:** 2022-10-14

**Authors:** Lidia Kuznetsova, Elizabeth Diago-Navarro, Rachel Mathu, Antoni Trilla

**Affiliations:** 1 Barcelona Institute for Global Health (ISGlobal), Hospital Clínic, Universitat de Barcelona, 08036 Barcelona, Spain; 2 Columbia University Irving Medical Center, New York, NY 10032, USA

**Keywords:** COVID-19, vaccination policy, mandatory vaccination, vaccine incentives, vaccine uptake, Europe

## Abstract

During 2021–2022 many countries in the European region of the World Health Organization (WHO) adopted mandatory and incentive-based vaccination measures to stimulate immunization against COVID-19. The measures ranged from positive incentive-based programs (i.e., cash incentives, meal discounts, and lotteries) to introducing COVID-19 certificates and enforcing the universal mandatory vaccination with fines. We assessed the effect of such interventions on COVID-19 vaccine uptake in the population of eight countries within the region. An interrupted time series (ITS) analysis was performed using an autoregressive integrated moving average (ARIMA) approach to account for autocorrelation and seasonality. The results showed the immediate positive impact of vaccination incentives on vaccine uptake in most cases, with the highest impact being cash incentives for the population (1197 per million population per day). Discount incentives did not show any significant impact. The introduction of COVID-19 certificates was associated with a significant immediate or gradual increase in daily administered vaccine doses in all the countries included in the study, up to 117,617 doses gained per million per month. The effect of mandatory vaccination for all or some groups of the population varied from a continuous decrease in daily administered doses (332 per million capita per day), no significant effect, or a delayed or temporary increase (1489 per million capita per day).

## 1. Introduction

The COVID-19 pandemic showed that the significance of the topic of vaccines and vaccination policies extends beyond the public health domain [1]. COVID-19 vaccination policies have been implemented during a period of heightened global tension and a systemic crisis, and some of them fueled political turbulence in a number of countries [2]. The choice of vaccination policy can make a profound effect on peoples’ life and society [3,4]. Therefore, thorough selection and application of the correct vaccination policy is of paramount importance [5]. This requires comprehensive evidence on what works and what does not in different settings. Such knowledge should be translated into interventions that maximize the benefits and minimize the harm for the population. 

As the pandemic persists, vaccination policies will continue to play an important part in mitigating the potential waves of COVID-19. Therefore, more evidence is needed on the effectiveness and impact of past and current vaccination policies in addition to updated vaccines [6]. Not only is the exploration on the impact of vaccination policies important for evaluation of their efficacy, it aids in the development of new policies to prepare for future public health crises [7]. The knowledge gained is not relegated solely to prevent the spread of COVID-19, but can be used to prevent disease spread for other multi-country outbreaks such as monkeypox, declared a Public Health Emergency of International Concern by the WHO, and hepatitis [8,9]. An increase in the incidence of vaccine-preventable diseases, triggered by vaccine hesitancy and aggravated by disruptions of immunization services due to the COVID-19 pandemic, are additional threats that may be mitigated with the addition of evidence-based knowledge surrounding vaccine mandates and incentives [10,11].

There is a lack of comprehensive and systematic data regarding the effectiveness of mandatory and incentive-based vaccination schemes; even when available, data are often not adequately utilized or examined. During the COVID-19 pandemic, some countries adopted unprecedented measures to increase vaccine uptake, such as lockdowns and limited access to businesses for the unvaccinated. Despite some studies that explored the impact of the implementation of country-level measures during the 2021 time period, such as the introduction of COVID-19 vaccine certificates, more evidence is needed to understand the impact of the whole range of interventions aimed at increasing vaccine uptake [12]. Focus drawn to understanding the effects of both announcement and enforcement of interventions, when feasible, is considerable to understanding the full picture of vaccination policies and implementation. 

During 2021–2022, many countries in the European region of the WHO, which comprises 53 countries, adopted mandatory and incentive-based vaccination measures to stimulate the immunization against COVID-19. The measures introduced in the countries of the region range from positive incentive-based approaches (i.e., cash incentives, meal discounts and lotteries) to introducing COVID-19 certificates or enforcing the universal mandatory vaccination with fines.

This article addresses one of the most pressing social challenges of mandatory vaccination. The aim of the study is to assess the effect of mandatory and incentive-based vaccination measures on vaccine uptake for COVID-19 in the population of the countries within the WHO European region. The study focuses on eight countries (Austria, Greece, Italy, Norway, Poland, Russia, Spain, and the United Kingdom) that introduced one or more of such measures at a specific point during the 2021/2022 time period, and where data were available pre- and post-intervention. The included countries have varied levels of COVID-19 vaccine acceptance, from high (Norway, Spain, the UK) to low (Poland and Russia) [13,14,15].

## 2. Methods

### 2.1. Policy Interventions

The interventions introduced between June 2021 and January 2022 in eight countries of the WHO European region (Austria, Greece, Italy, Norway, Poland, Russia, Spain, and the United Kingdom) were analyzed. The list of the analyzed policy interventions in each country is presented in Table 1. Wherever feasible—depending on data availability, availability of information about the date of announcement and enforcement of measures, sufficient time points between interventions—we assessed the effects of both the announcement and enforcement of each intervention. For Greece, Poland, Russia, and the UK, the following announcements/enforcements were analyzed: cash incentives, lotteries, municipalities competitions, and discount vouchers. The impact of COVID-19 certificates was assessed for Greece, Norway, Russia, and Spain. Lastly, the following interventions were studied for Austria, Greece, Italy, and Russia: lockdown for the unvaccinated, mandates for adults aged 50/60 and older, mandates for the employees, and the universal vaccine mandate.

### 2.2. Data Sources

Data on daily administered COVID-19 vaccine doses were sourced from Our World in Data, which utilized governmental reports to inform their database [16]. Additionally, we consulted official governmental sources in cases of missing data or in the case of Russia, where Our World in Data did not use official data provided by the Russian government, but the data from alternative sources. Furthermore, we referred to the European Centre for Disease Prevention and Control COVID-19 Vaccine Tracker for data for specific age groups for the relevant interventions in the European Union countries [17]. The dates of policy interventions were obtained from official governmental documents, local and international news outlets. 

### 2.3. Population

The study focuses on the population of eight countries of the WHO European region that introduced one or more of mandatory or incentive-based measures at a specific point in time, and where sufficient data were available before and after the introduction of the measures and on exact dates of introduction of the interventions, necessary for an ITS analysis with daily data. The rationale for our choice was to include countries that represent different levels of the COVID-19 vaccine acceptance. The included countries had the following levels of acceptance of the COVID-19 vaccine: high (Norway, Spain, UK), medium (Austria, Greece, Italy), and low (Poland, Russia) [13,14,15]. We also took into consideration whether the countries and interventions had been already assessed in other available studies, to avoid duplication. 

### 2.4. Outcome

The primary outcome of this analysis was the change in the number of daily administered doses following the interventions. Outcomes could be broken down into categories. An instantaneous impact is defined as an impact that occurs in the same period in which the policy is introduced, and the impact in the days following the introduction of the policy, that is, the dynamic effect of the impact. We also report the change in the number of administered vaccine doses per million capita, to facilitate the comparison between the interventions and countries. Through conducting a counterfactual analysis, we report the values of daily administered doses in the absence of interventions, and number of doses gained (or missed) due to the implementation of interventions. 

### 2.5. Statistical Analysis

An Interrupted Time Series, a quasi-experimental method, was used to carry out this analysis. ITS analyses have increasingly been applied to evaluate the impact of large-scale health interventions. The choice of this method was based on its suitability for the dataset, which satisfied the following conditions: clear differentiation of the pre-intervention period and the post-intervention period and sufficient number of datapoints. In their ITS regression tutorial Bernal et al. argue that ITS works best with short-term outcomes that are expected to change either relatively quickly after an intervention is implemented or after a clearly defined lag [18]. According to Schaffer et al., the ITS method is suitable for the evaluation of ‘natural experiments’ implemented in real-world settings and is becoming widely used with the increasing availability and quality of routine data spanning before and after interventions [19]. The ITS analysis has been conducted through the following steps: (1) proposing an impact model, that is assuming whether the change following the intervention is immediate or gradual and whether there will be a lag period before any effect is expected. (2) Conducting descriptive analysis, consisting of initial summary statistics and plots to explore the data. (3) Carrying out regression analysis. Such analysis can be performed through segmented regression, which requires a linear or easily modelled trend and independently distributed residuals. Segmented regression was not suitable for out data, due to the difficulty in modelling the autocorrelation structure. Therefore, we used ARIMA approach to account for autocorrelation and seasonality. (4) Addressing methodological issues, such as seasonality, autocorrelation and confounders. (5) Model checking [18]. When the assumption of constant variance was not met for the ARIMA model, other models were explored. These included autoregressive conditional heteroskedasticity (ARCH) and generalized autoregressive conditional heteroskedasticity (GARCH) models [20]. For Norway, the use of the autoregressive distributed lagged model (ARDL) was explored, as ARIMA models failed to provide a good fit for the data.

Furthermore, we conducted a counterfactual analysis to obtain the values predicted by the ARIMA model in absence of the intervention compared with the observed values. To do this, we built additional ARIMA models for the periods up to the time before the intervention, with the forecasts representing what would happen if no intervention had been put into place.

To develop the models, the general to particular approach was applied. This consisted of starting from a dynamic model with a large lag structure (12, 24, or 30 days), and then eliminating the non-significant lags until a nested model with all significant variables was identified. The intervention variables were entered as predictors into the model, and if statistically significant, this induced a positive or negative change in the intercept of the model.

Stationarity was tested by Dickey–Fuller test, and autocorrelation by the Ljung–Box test. Autocorrelation, nonstationarity, and seasonality were handled through differencing in the ARIMA model. To check the stability of the models, the calculation of the inverse values of the roots of the autoregressive polynomial was performed. To conduct the statistical analysis, Eviews (version 12; IHS Markit, London, UK) and SPSS software (version 28.0; IBM Corp., Armonk, NY, USA) were utilized. 

## 3. Results

The data were analysed by country and type of implemented measure.

### 3.1. Austria

The sample consisted of 161 observations for the period 1 September 2021–8 February 2022. The mean number of daily doses in the sample was 44,984, while 50 percent of the observations were below 32,736 doses. The maximum number of doses was 112,599, and the minimum number of doses was 12,932.

**Figure 1:**
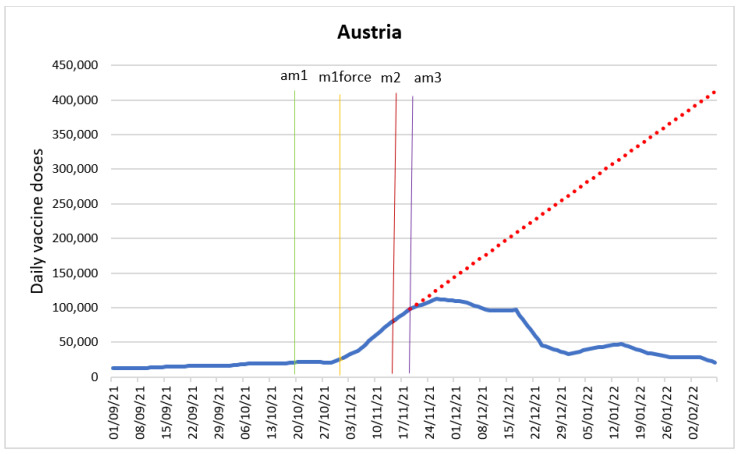
Daily new vaccinations in Austria before and after the policy changes and predicted values in absence of intervention based on ARIMA model. 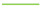 Announcement of employees vaccine mandate (am1). 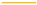 Employees mandate in force (m1force). 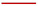 Lockdown for unvaccinated (m2). 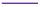 Announcement of universal mandate with fines (am3). 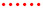 Predicted values in absence of announcement of the universal vaccine mandate (am3).

The purpose of the ARIMA model was to determine the impact of the following intervention variables: announcement of the employee vaccine mandate (am1), enforcement of the employee vaccine mandate (m1force), introduction of lockdown for the unvaccinated (m2) and announcement of the universal vaccine mandate with fines (am3). Figure 1 shows the time series with the intervention time points.

The ARIMA model shows that the only intervention variable affecting the doses variable was am3, the universal vaccine mandate with fines, which was statistically significant at any significance level (*p*-value < 0.000). The announcement of the universal vaccine mandate (am3) acted as a leading indicator, that is, it impacted doses administered one day after the announcement. This announcement on 19 November 2021 had a negative impact on doses and was associated with a reduction from on intervention period to the next by 3010 doses (95% CI: −3592–2428). The introduction of mandatory vaccination for the employees (am1) did not produce an immediate effect but likely produced an anticipatory and/or delayed effect. This is in line with the data on vaccine coverage among different age groups (17). The observed effect can also be linked to the data reporting issues for Austria. In order to produce a complete series of daily figures and account for missing data Our World in Data, the data source utilized, used a rolling 7-day average window, possibly hiding some of the significant effects of the assessed interventions [21]. As for the lockdown for the unvaccinated, which was in force for only 4 days, the lack of any significant effect can be linked to the short duration of the intervention. Finally, the negative effect of the introduction of the universal vaccine mandate (am3) is likely to be due to the substitution on 19 November 2021 of the lockdown for the unvaccinated (m2) (announced just 5 days before on 15 November) with vaccine mandate for everybody with the fines applied from the next year and the immediate lockdown for everybody. These quick changes resulted in the unvaccinated losing the motivation to vaccinate, which was likely tied to the general confusion felt among the Austrian population due to rapidly changing measures and policy unpredictability [22]. As a result, it is likely that many preferred to adopt the “wait and see approach”. These findings are in line with the results of an initial comparison of vaccination policies and vaccine uptake between Austria and Germany in November to December 2021 [23].

It was necessary to introduce three dummy variables in the periods 30 October 2021, 18 December 2021 and 25 December 2021 to account for an 11.51% increase or 1510 doses (95% CI: 1078–1941), a 5.52% drop and a 3.98% drop in doses, respectively. These dummies correct for atypical behaviors in the dose variable. The October increase is associated with the entry into force of the employee vaccine mandate on 1 November 2021, whereas the December outliers are related to the holiday season. 

Figure 1 shows the forecast of the evolution of the daily doses in the absence of intervention based on ARIMA model. The forecast has been generated in relation to am3 intervention, since it was the only intervention variable that produced a statistically significant result. The forecast shows that if there was no substitution of the lockdown for the unvaccinated (m2) with the announcement of the universal mandate (am3) together with the immediate lockdown for everybody, the vaccine uptake would have grown due to the implementation of the lockdown for the unvaccinated (m2). This forecast assumes an infinite number of people to vaccinate, which can be considered its limitation.

### 3.2. Greece

The sample included 273 observations covering the period 1 June 2021–28 February 2022. The mean number of daily doses was 53,884, with 50 percent of the observations below 45,991 doses. The maximum number of doses was 109,105 and the minimum number of doses was 18,105.

**Figure 2:**
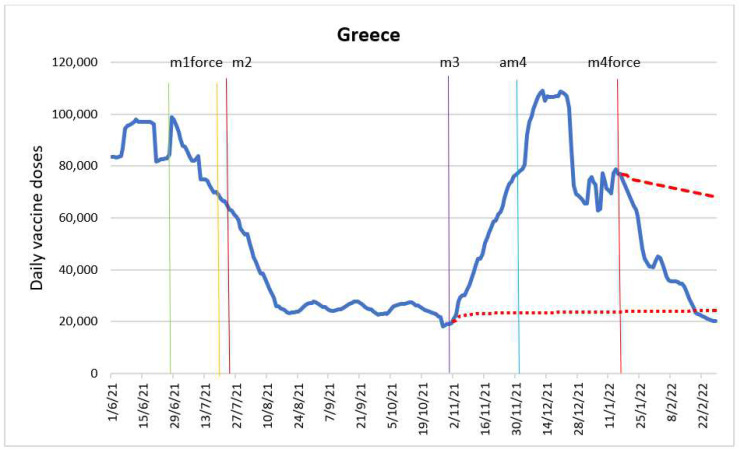
Daily new vaccinations in Greece before and after the policy changes and predicted values in absence of interventions based on ARIMA models. 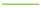 Announcement of “freedom pass” (am1) 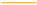 Introduction of “freedom pass” (m1force) 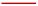 Financial incentives for pharmacists and doctors (m2) 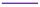 Introduction of COVID certificates (m3) 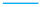 Announcement of vaccine mandates for adults aged 60 and older (am4) 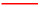 Vaccine mandate for adults aged 60 and older in force (m4force) 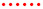 Predicted values in absence of COVID certificates 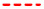
Predicted values in absence of entry into force of the vaccine mandate for adults aged 60 and older.

The purpose of the ARIMA model was to determine the impact of the following intervention variables: introduction of “freedom pass” (m1force), introduction of financial incentives for pharmacists and doctors (m2), introduction of COVID-19 certificates (m3), entry into force of the vaccine mandate for adults aged 60 and older (m4force), announcement of entry into force of “freedom pass” (am1), and announcement of entry into force of the vaccine mandate for adults aged 60 and older (am4). Figure 2 shows the time series with the intervention time points.

The intervention variables m1force and am4 were not significant. According to the estimated model, the announcement of the entry into force of the “freedom pass” (am1) on 28 June 2021, had an immediate and delayed impact. Meaning in the current period it increased the number of interperiod doses by 12,354 (95% CI: 11,755–12,954); however, one period after the announcement it indicated a drop of 14,715 (95%CI: −15,196–14,235) doses period to period. Further, the announcement of the entry into force of the “freedom pass” (am1) led to a drop of 2361 doses interperiod. This is in line with data on vaccine coverage in the age group targeted by the introduction of the “freedom pass” [17]. Regarding financial incentives for pharmacists and doctors (m2), this impacted the number of doses two days after its entry into force on 23 July 2021, implying an interperiod increase of 1546 (95% CI: 1215–1878) doses. Entry into force of COVID-19 certificates (m3) on 1 November 2021 had an immediate impact, increasing the number of doses interperiod by 1500 (95% CI: 1284–1716) doses. Finally, the entry into force of the vaccine mandate for adults aged 60 and older (m4force) on 17 January 2022 had a negative impact on the same period, resulting in a fall of 1932 (95% CI: −2066–1798) doses period to period. Authors argue that there was a positive impact of the announcement of vaccine mandates for people over 60, as the number of those who received the first dose of COVID-19 vaccine increased substantially by the end of 2021 [24].

Figure 2 shows the forecast of the evolution of doses in the absence of the introduction of COVID-19 certificates (m3) based on an ARIMA model. It illustrates a substantial impact of the introduction of COVID-19 certificates (m3) on vaccine coverage in the country. The figure also shows the forecast of the evolution of the daily doses in the absence of entry into force of the vaccine mandate for adults aged 60 and older (m4force), based on ARIMA model. The measure (m4force) entered into force in the final stage of the mass vaccination campaign, which was not accounted for in the forecast, and did not induce any increase in the administered doses, as there were very few individuals left in the population to be vaccinated. This intervention was clearly aimed at the few people who resisted vaccination the most.

### 3.3. Italy

The sample of 181 observations covered the period 1 September 2021–28 February 2022. The average number of daily doses in the sample was 312,657, while 50 percent of the observations were under the daily dose level of 248,011. The maximum number of doses was 665,777, while the minimum number was 114,725.

**Figure 3:**
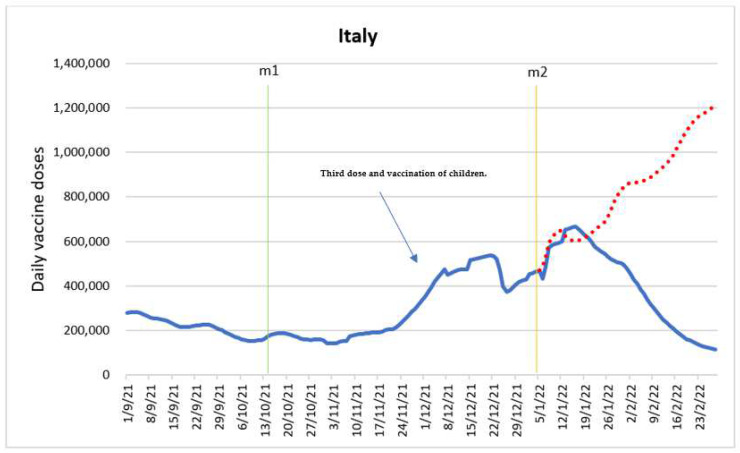
Daily new vaccinations in Italy before and after the policy changes and predicted values in absence of intervention based on ARIMA models. 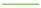
Introduction of “Green pass” mandate for employees (m1) 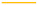 Introduction of vaccine mandate for adults aged 50 and older (m2) 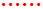 Predicted values in absence of entry into force of the vaccine mandate for adults aged 50 and older.

The main reason for estimating the model was to determine the impact of intervention variables, specifically, the entry into force of the “Green pass” mandate for employees (m1) and the entry into force of the vaccine mandate for adults aged 50 and older (m2). Figure 3 shows the time series with the intervention time points. 

According to the estimated model, only the vaccine mandate for adults aged 50 and older (m2) had a significant impact on the number of administered vaccine doses. This is in line with the data on vaccine coverage among different age groups (17). The model shows that m2 impacted with lags. In fact, once m2 came into effect on 5 January 2022, the impact on the number of doses took place from the following period, inducing an interperiod drop of 43,357 (95% CI: −53,057–33,659) doses, to two periods beyond the effective date where there was a change in the trend. This change resulted in an increase of 89,728 (95% CI: 76,848–102,610) doses from period to period. The direction of the trend was maintained for one more period, but with a smaller interperiod growth of 17,919 (95% CI: 8321–27,518) doses. Four periods after the entry into force of m2, the measure’s previous trend was reversed, implying an interperiod drop of 76,621 (95% CI: −86,530–66,712) doses. Further, the impact of m2 on daily dose-behavior was negative, indicating an interperiod decrease of 12,330 doses on the fifth period. 

A substantial increase in the number of administered doses in the period between the end of November and Christmas holidays can be attributed to the mass administration of the third dose of the vaccine, as well as the start of the vaccination of 5–11-year-old children [25,26].

Figure 3 shows the forecast of the evolution of the daily doses in the absence of entry into force of the vaccine mandate for adults aged 50 and older (m2), using an ARIMA model. The forecast assumes an infinite number of people to vaccinate. The discrepancy between the observed values and long-term forecast has similarities to the projected Greece forecast where the vaccine mandate for adults aged 60 and older is introduced during the final period of the mass vaccination campaign with few people left to vaccinate.

Several published studies have analyzed COVID-19 vaccination mandates in Italy [12,27,28]. However, their focus was on the effect of the introduction of COVID-19 certificates in Italy in July 2021, which was not a period covered by our data. The results indicated that this intervention was associated with a significant increase in administered first doses of COVID-19 vaccine in Italy. Our findings provide insights on further developments in the vaccination campaign in Italy.

### 3.4. Norway

The sample of 182 observations spanned the period 2 June 2021–30 November 2021. The mean number of daily doses was 32,630, and the number of doses in which 50 percent of the observations fell below was 35,443. The maximum number of doses was 69,114, while the minimum was 4762.

As the attempted ARIMA models did not appropriately fit the data, we estimated an autoregressive distributed lagged model, where the dependent variable entered as a significant lagged explanatory variable until period 7. It was necessary to introduce four dummy variables during the sample period (7 July 2021, 31 August 2021, 1 September 2021 and 22 September 2021) to pick up outlier effects. These dummy variables were associated with a decrease in doses of 7.11%, 3.55%, 9.01%, and 12.05%, respectively. The outliers are related to summer holidays and COVID-19 vaccination calendar in Norway. The vaccination calendar is important to note during the period of our sample as it coincides with the earlier stages of the mass vaccination campaign in Norway, where the numbers of daily doses were dependent on vaccination appointments offered to people of certain age groups [29].

**Figure 4:**
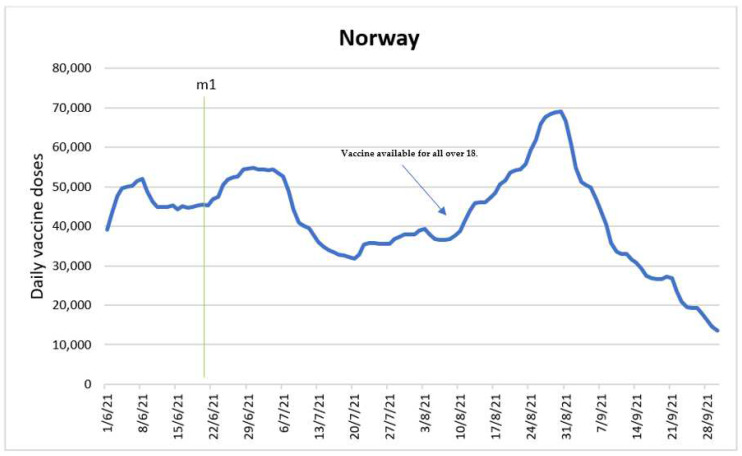
Daily new vaccinations in Norway before and after the policy change. 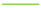 Introduction of COVID certificates (m1).

The purpose of the ARDL model was to determine the impact of a single intervention variable, which was entry into force of the COVID-19 certificates (m1). Figure 4 shows the time series with the intervention time point. 

The entry into force of COVID-19 certificates was on 20 June 2021 and remained in force until 25 September 2021. According to the estimated ARDL model, once the measure came into effect it had a positive impact after four days, with an approximate increase of 1308 doses from one period to the next, and a negative impact of 1399 doses on the fifth day after coming into effect. Further, the effect is negative, with a drop from one period to another of 90 doses (1308.2 + (−1398.64)). This period overlaps with the availability of vaccinations for the 45–54 age group, according to the vaccination calendar. From August 2021 COVID-19 vaccine became available for everybody over 18 years old in Norway, and the summer holidays in ended the same month, which is reflected in the spike of the vaccine uptake curve [29]. Therefore, the introduction of COVID-19 certificates in Norway was likely to produce moderate supporting effect to motivate people to vaccinate.

### 3.5. Poland

The sample comprised 122 observations for the period 1 June 2021–30 September 2021. An average number of 145,891 daily doses were observed in the dataset, while 50 percent of the data fell below 99,022 daily doses. The strong discrepancy between the mean and median suggests the existence of outliers in this dataset. The highest dose level observed was 341,329, while the lowest dose level was 19,980.

**Figure 5:**
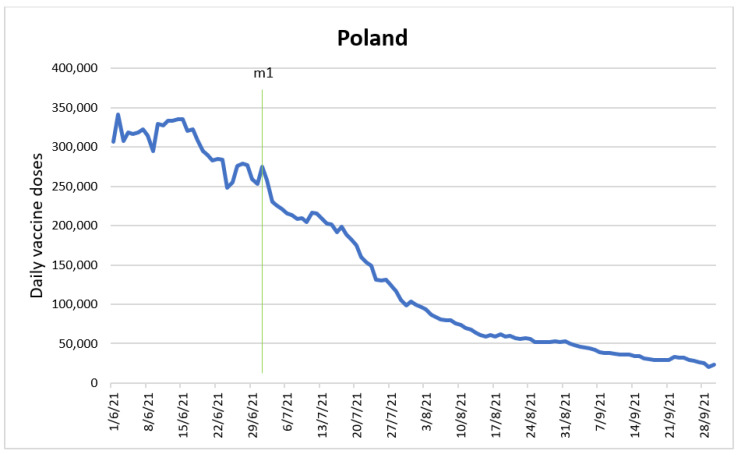
Daily new vaccinations in Poland before and after the policy change. 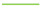 Start of national lottery and municipality competitions (m1).

An ARIMA GARCH model was developed to determine the impact of the intervention variable “introduction of national lottery and municipality competitions” (m1). Figure 5 shows the time series with the intervention time point.

When the measure came into force on 1 July 2021, it had a positive impact with an interperiod increase of 25,196 doses. One period after the measure’s entry into force, the trend shifted and resulted in a decrease of 24,368 doses from period to period. Further, the measure produces an interperiod increase of 827 doses (25,196.74 + (−24,368.91)).

It was necessary to introduce dummy variables on the dates 24 June 2021, 26 June 2021, 3 July 2021 and 11 July 2021 to pick up irregularities in the dependent variable. These irregularities were a drop of 12.47%, an increase of 8.46%, a fall of 10.65% and an increase of 5.61%, respectively. The variations can be explained by the vaccination schedule and logistical reasons, such as closure of vaccination points [30].

The intervention had an immediate temporary positive effect on the number of daily administered vaccine doses. To our knowledge, there are no published studies evaluating this initiative specific to Poland; however, there are studies that examined the effect of incentives on vaccine coverage in other countries such as the USA. Our findings are in line with the conclusion derived by these researchers, reporting an increase in COVID-19 vaccinated rates after the implementation of conditional cash lotteries in Ohio [31]. Despite the abundance of COVID-19 vaccines in Poland, there was an overall decrease in the weeks after the implementation of the intervention due to vaccine hesitancy among the population [30]. The summer holidays season also contributed to the general reduction [32]. 

### 3.6. Russia

The sample consisted of 255 observations for the period 1 June 2021–10 February 2022. The average number of daily doses was 502,514, with a median of 484,198 daily doses. The maximum number of doses was 1,020,075 and the minimum number of doses was 66,419.

**Figure 6:**
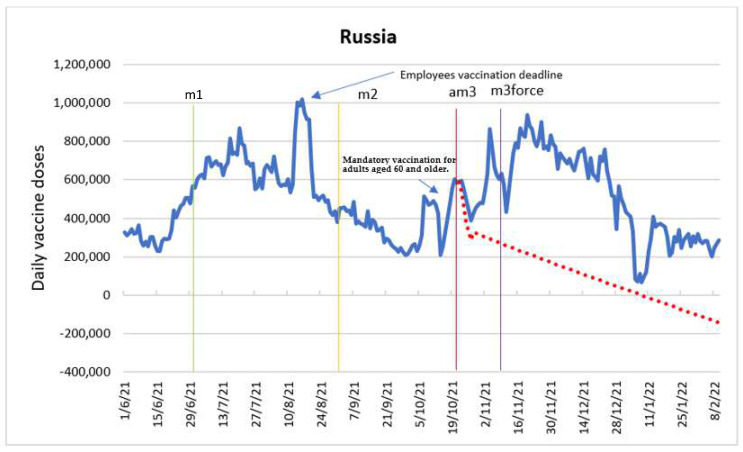
Daily new vaccinations in Russia before and after the policy changes and predicted values in absence intervention based on ARIMA models. 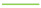 Introduction of employees vaccine mandate (m1) 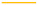 Lottery for the vaccinated (m2) 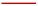 Announcement of the introduction of COVID certificates (am3) 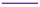 COVID certificates in force (m3force) 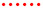 Predicted values in absence of the announcement of COVID certificates.

The purpose of the ARIMA model was to determine the impact of intervention variables, namely: introduction of employee vaccine mandates (m1), launch of the lottery for the vaccinated (m2), announcement of the introduction of COVID-19 certificates (am3) and entry into force of COVID-19 certificates (m3force). Figure 6 shows the time series with the intervention time points and the forecast of the evolution of the daily doses in the absence of the announcement of COVID-19 certificates (am3), based on the ARIMA model. We made the forecast for am3 as it was statistically significant, and the dataset contained appropriate number of pre- and post-intervention datapoints to develop an ARIMA model.

According to the estimated ARIMA model, the intervention associated with the entry into force of COVID-19 certificates (m3force) was not significant. The entry into force of employee vaccine mandate (m1) on 1 July 2021 had an immediate but negative impact on the number of doses; stimulating a drop from one period to another by 43,429 doses. On the other hand, the launch of the lottery program (m2) in the period 1 September 2021 had an immediate positive impact, increasing the number of doses interperiod by 9766 doses. The announcement of the entry into force of COVID-19 certificates (am3) on 22 October 2021 had an immediate and lagged impact on the behavior of doses. The immediate impact was negative, with a fall from one period to another in the order of 42,718 doses. In the period after the announcement, the impact was positive with an interperiod increase of 43,775 doses. Further, am3 implied an interperiod increase of 1056 doses.

Even though the analysis showed an immediate negative effect of the introduction of the employee vaccine mandate, the intervention produced a positive delayed effect. Russia’s vaccination curve reflects the regulation implemented in many regions of the country, according to which the employers must vaccinate with the first dose 60% of the employees by the middle of July 2021 and with the second dose by the middle of August 2021 [33,34]. This measure was accompanied by additional supply of COVID-19 vaccines to the regions and setting up additional vaccination points [35].

An increase in the number of administered doses in the middle of October and the beginning of November 2021 coincides with the introduction of mandatory vaccination for adults aged 60 and older in a subset of regions throughout the country [36,37]. Financial incentives to doctors and nurses for vaccination accompanied this measure [38]. It was not feasible to analyze the effect of these measures as they were introduced gradually and did not cover all the regions.

Finally, the increase in the vaccination in December 2021 fell at the time of when many Russians were preparing for the two-week long winter holiday at the end of December. Many travelled during that period, and were required to have a COVID-19 certificate to access resorts and other facilities within the country. As a result, this increase can be considered a delayed effect of the introduction of COVID-19 certificates [39].

### 3.7. Spain

The sample consisted of 163 observations for the period 1 September 2021–10 February 2022. There were no missing values during this time period. The average number of daily doses was 157,899, and the number of doses below which 50 percent of the observations were found was 146,011. The minimum number of doses observed was 2724, while the maximum number of doses was 670,109.

**Figure 7:**
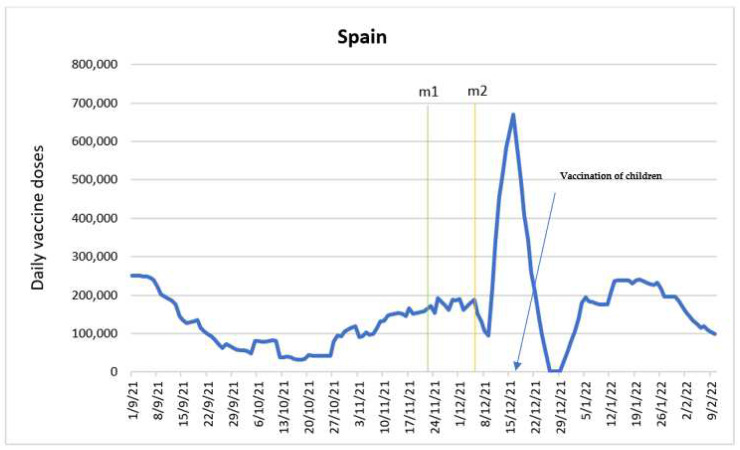
Daily new vaccinations in Spain before and after the introduction of COVID certificates. 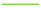 Announcement of the introduction of COVID certificates (m1) 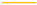 COVID certificates in force (m2).

The main purpose in estimating the ARIMA model was to determine the impact of the predictor variables: announcement of the introduction of COVID-19 certificates (m1) and entry into force of COVID-19 certificates (m2). Figure 7 shows the time series with the intervention time points.

Regarding the impact of the variable m1, on the date when the announcement of the future entry into force of the regulation was made in most of the regions that applied COVID-19 certificates (22 November 2021), the impact was positive, producing an increase of about 24,902 doses. However, in the three days following that initial increase, the positive effect vanishes. The first day resulted in a decrease of 19,015, followed by a decrease in the order of 32,626 doses three days after the initial spike. In the three days following the announcement, and positive spike, there was a cumulative decrease in the number of doses of 51,641 on average. 

Enforcement of the COVID-19 certificates (m2) had a negative impact on the date the regulation came into effect, inducing a decrease of 61,287 doses on average. However, in the three days following the introduction the impact of the measure became positive. An increase of 49,711, 58,830, and 103,764 doses were seen on the three days following its introduction, respectively. This can also be interpreted as a cumulative increase of about 212,306 doses over this three-day period. Estimates show that the positive impact of the regulation’s entry into force was greater than its announcement; however, the positive impact of the implemented regulation acted as a leading indicator. 

Two additive outliers were found on 12 October 2021 and 26 December 2021 that negatively impacted the dose variable, causing decreases of 16,295 and 14,139 doses, respectively. These outliers coincide with holidays in Spain. A transitory outlier was also identified on 16 December 2021 causing a positive impact, increasing the number of doses by 41,090. This increase corresponds to the start of the vaccination of 5–11 year old children; the estimated decay factor was 0.445 [40].

The positive effect of the introduction of COVID-19 certificates on the number of administered doses in Spain is evident. The regulation was introduced in the context of the preparation for Christmas holidays and the spread of Omicron variant. Furthermore, to facilitate the vaccination, the authorities set up additional vaccination points in this period [41]. COVID-19 certificates increased vaccination numbers beyond the regions where they were introduced due to increased travel of the population during the holiday season [42]. A number of other countries implemented COVID-19 certificates, and several studies analyzing these measures implemented in summer 2021 have been published. Our findings are in line with the results delivered by these studies, reporting a significant increase in COVID-19 vaccine uptake in Canada, France, Germany, Israel, Italy, and Switzerland [12,27,28]. 

### 3.8. United Kingdom

The sample consisted of 92 observations for the period 1 July 2021–30 September 2021. The mean number of daily doses in the sample was 180,283, with 50 percent of the observations falling below 200,182. The maximum number of doses in the sample was 327,284, while the minimum number was 63,283 doses.

**Figure 8:**
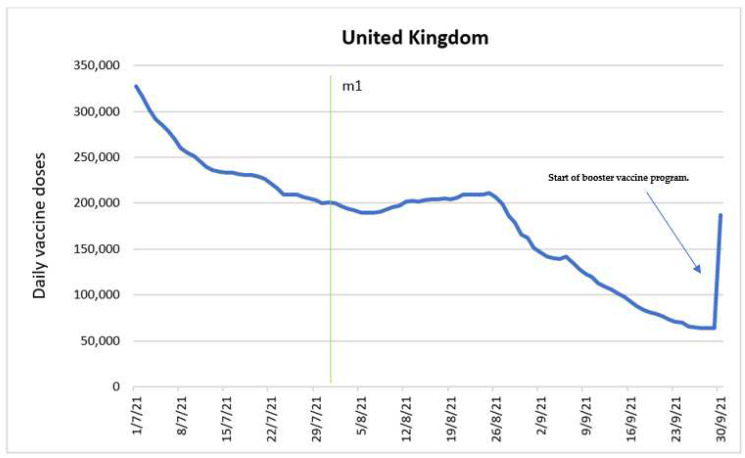
Daily new vaccinations in the United Kingdom before and after the introduction of discounts and vouchers for the vaccinated. 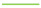 Introduction of discounts and vouchers for the vaccinated (m1).

An ARIMA ARCH model was developed to determine the impact of the intervention variable “introduction of discounts and vouchers for the vaccinated” (m1) on the number of doses administered. Figure 8 shows the time series with the intervention time point.

According to the ARIMA ARCH model, the intervention variable impacts the number of doses one period after its entry into force on 1 August 2021. However, this impact was not statistically significant (*p* = 0.1345), which is higher than the standard significance levels of 1%, 5% or 10%. Regardless, the effect on the number of doses is negative, inducing a fall of 2558 doses period to period.

It was necessary to introduce dummy variables on the dates 5 July 2021, 31 August 2021 and 7 September 2021. These dates correspond to outliers were doses decreased by 1.99%, 2.31%, and 4.73%, respectively. It is likely that these drops are related to seasonal holidays. The increase in vaccinations at the end of September 2021 coincides with the start of a COVID-19 booster vaccine program in the country [43].

The lack of a significant effect of the introduction of discounts and vouchers on the number of vaccinations is in line with the data on vaccine coverage in the age group targeted by the intervention designed to boost the vaccine uptake among adults under 30 [44].

Table 2 summarizes the results that proved to be statistically significant.

## 4. Conclusions

We examined the effect of mandatory and incentive-based vaccination measures on vaccine uptake for COVID-19 in the population of eight countries within the WHO European region. Our findings show the immediate positive impact of vaccination incentives on vaccine uptake in most cases, with the highest impact of cash incentives for the population seen in Greece (1197 per million population per day). Discounts incentives did not show any significant impact. The introduction of COVID-19 certificates was associated with a significant immediate (Spain) or gradual increase (Greece) in daily administered vaccine doses in all the countries included in the study, up to 117,617 doses gained per million per month seen in Russia. The effect of mandatory vaccination for all or some groups of the population varied from a continuous decrease in daily administered doses (Austria—332 per million capita per day), no significant effect, delayed or temporary increase (Italy—1489 per million capita/day). Our study provides new insights on the effectiveness of vaccine interventions that had not previously been assessed. The impact of both incentive and mandate interventions are of great interest to various stakeholders including researchers, policy makers, and the general population. The study has several limitations: it does not account for changes in the required vaccine doses over time, differences in the countries’ information campaigns aimed at increasing the vaccine uptake and differences in the degree of ethical concerns regarding mandatory vaccination, which was beyond the scope of this study. It is important to note that these findings should be applied with caution, especially in policy making. Interventions producing a significant effect on the vaccine uptake may come at a cost, both direct financial and indirect. Further research is needed in many additional areas and disciplines to have a complete understanding of the impact of mandates, other than its direct impact on doses. This includes evaluating the costs of implementation of vaccine incentives and mandates, assessing individuals’ willingness to trust authorities or vaccines, as well as the effect on civil liberties and collateral effects such as forging the evidence of immunization status. The COVID-19 pandemic has highlighted many inadequacies in public health response, but has also been a demonstration of ingenuity. It is obvious that high level of vaccine hesitancy is reflected in lower vaccine uptake. At the same time, we did not find clear difference in the effectiveness of the interventions among the countries with different levels of the vaccine acceptance. Yet, vaccination policies have varied impact depending on context and setting. This in itself is an important outcome because it gives countries the tools to learn from their own policies, as well as the policies of like nations. Moving forward, long-term programs such as scientific outreach activities should be ongoing to provide people with objective and transparent information and education regarding vaccinations in order to improve the outcomes of emergency public health initiatives in the future.

## Figures and Tables

**Table 1:** Summary of COVID-19 vaccination mandates and incentives in the selected countries.

Country	Measure Description	Announced	Introduced	Suspended	Selected Sources
Austria	Introduction of a general requirement for ‘3G’ proof to enter the workplace. The ‘3Gs’ are ‘geimpft, genesen oder getestet’ (abbreviated as 3G as all three words start with a G in German) meaning vaccinated, recovered or tested.	20 October 2021	1 November 2021	March 2022	https://www.thelocal.at/20220223/austria-to-lift-3g-requirement-for-workplaces/ (accessed on 15 August 2022)
	Lockdown for the unvaccinated. Unvaccinated people in Austria will be allowed to leave their homes only for work, food shopping or emergencies.	15 November 2021	15 November 2021	19 November 2021	https://www.bbc.com/news/world-europe-59343650 (accessed on 15 August 2022) https://www.npr.org/sections/coronavirus-live-updates/2021/11/15/1055839727/austria-and-germany-impose-restrictions-on-unvaccinated-people-as-covid-cases-su (accessed on 15 August 2022)
	Universal mandate with fines announced. Those over the age of 18 who decline to take a jab face penalty of up to 3600 EUR, unless they are pregnant or severely ill.	19 November 2021	February 2022	March 2022	https://www.theguardian.com/world/2022/mar/09/austria-suspends-mandatory-covid-vaccination-law (accessed on 15 August 2022) https://www.bnnbloomberg.ca/austria-suspends-vaccine-mandate-as-omicron-questions-policy-1.1734807 (accessed on 15 August 2022)
Greece	“Freedom pass”—150 EUR voucher offered to people 18–25 years old who get vaccinated.	28 June 2021	20 July 2021	31 December 2021	https://news.gtp.gr/2021/07/23/greece-pushes-covid-19-vaccination-new-incentives-laws/ (accessed on 15 August 2022) https://emvolio.gov.gr/freedompass-datapass (accessed on 15 August 2022)
	Financial incentives for pharmacists and doctors which include subsidies for booked vaccination appointments as well as for in-home vaccinations.	23 July 2021	23 July 2021		https://news.gtp.gr/2021/07/23/greece-pushes-covid-19-vaccination-new-incentives-laws/ (accessed on 15 August 2022)
	Those vaccinated against COVID-19 have to present their vaccination certificates, while unvaccinated people are required to present a negative COVID-19 test to enter all shops, banks and public indoor areas, as well as outdoor restaurants and cafes. Exceptions were made for food shops, pharmacies, and places of worship.	2 November 2021	6 November 2021	1 May 2022	https://abcnews.go.com/Health/wireStory/greece-toughens-restrictions-unvaccinated-cases-spike-80927971 (accessed on 15 August 2022) https://www.reuters.com/world/europe/unvaccinated-greece-face-new-restrictions-covid-cases-soar-2021-11-06/ (accessed on 15 August 2022) https://news.gtp.gr/2022/04/12/greece-suspend-covid-19-vaccination-and-recovery-certificates-next-month/ (accessed on 15 August 2022)
	Vaccination mandate for all residents of Greece over the age of 60. The unvaccinated would be fined 100 EUR for every month they remain unvaccinated.	1 December 2021	17 January 2022	15 April 2022	https://abcnews.go.com/Health/wireStory/greece-approves-mandatory-vaccination-aged-60-81500213 (accessed on 15 August 2022) https://www.bbc.com/news/world-europe-59474808 (accessed on 15 August 2022) https://greekcitytimes.com/2022/04/15/over-60s-in-greece-ends-as-of-today/ (accessed on 15 August 2022)
Italy	Workers cannot access any place of work in Italy without a ‘Green Pass’, which is issued if the holder has been fully vaccinated against COVID-19, received a negative result from a rapid or antigen test in the previous 48 h or recovered from COVID-19 in the previous six months.	September 2021	15 October 2021	May 2022	https://www.theguardian.com/world/2021/oct/14/italy-braced-for-unrest-as-covid-pass-becomes-mandatory-for-all-workers (accessed on 15 August 2022) https://www.gazzettaufficiale.it/eli/id/2022/03/24/22G00034/sg (accessed on 15 August 2022) https://www.reuters.com/world/europe/italy-readies-law-make-covid-health-pass-mandatory-all-workers-2021-09-16/ (accessed on 15 August 2022)
	Italy extends COVID-19 vaccine mandate to everyone over 50. The fine for non-compliance is 100 EUR.	5 January 2022	5 January 2022	15 June 2022	https://www.reuters.com/world/europe/italy-make-covid-jab-mandatory-over-50s-tighten-curbs-draft-2022-01-05/ (accessed on 15 August 2022) https://www.normattiva.it/uri-res/N2Ls?urn:nir:stato:decreto.legge:2022-01-07;1 (accessed on 15 August 2022) https://www.ilsole24ore.com/art/in-arrivo-nuova-stretta-anti-omicron-obbligo-vaccino-gli-over-60-AEOd2T6 (accessed on 15 August 2022)
Norway	Domestic use of COVID-19 certificates for coastal cruises and events, as well as authority for municipalities to regulate, e.g., opening of closed businesses using COVID-19 certificates.		20 June 2021	25 September 2021	https://www.regjeringen.no/no/dokumenter/prop.-10-l-20212022/id2880813/?ch=5 (accessed on 15 August 2022)
Russia	Mandatory vaccination for employees in a wide range of sectors, covering most of employees in the country.		1 July 2021	May 2022	https://abcnews.go.com/Health/wireStory/russian-regions-make-vaccines-mandatory-workers-78334612 (accessed on 15 August 2022) https://www.loc.gov/item/global-legal-monitor/2021-06-22/russian-federation-unvaccinated-employees-can-be-suspended-without-pay/ (accessed on 15 August 2022)
	Lotteries for the vaccinated. Four draws of total 2000 cash prizes, 100,000 RUB each.		1 September 2021	30 November 2021	https://xn--80abehgib9bifaxh1a8l.xn--p1ai/ (accessed on 15 August 2022)
	Introduction of COVID-19 certificates in most regions, nationwide digital passes showing proof of vaccination or recent recovery from COVID-19 to enter public areas and events.	22 October 2021	8 November 2021	March 2022	https://www.themoscowtimes.com/2021/11/02/russia-imposes-digital-covid-passes-amid-virus-surge-a75461 (accessed on 15 August 2022)
Poland	Lottery for the vaccinated. Every day from July 1, every 500th participant to receive a cash prize of PLN200 (222 EUR) while every 2000th person was to win PLN500 and every week for three months until the end of September, the government gave several automobiles, five prizes of 50,000 PLN and 720 electric scooters. The government engaged municipalities all over Poland by launching a competition with numerous financial prizes. The commune with the highest percentage of the vaccinated will receive 2 million PLN. The first 500 municipalities that will vaccinate 67 percent will receive 100,000 PLN.		1 July 2021	30 September 2021 (lottery)	https://www.intellinews.com/poland-kicks-off-covid-19-vaccination-lottery-214644/?source=poland (accessed on 15 August 2022)https://www.gov.pl/web/loteria/zasady-i-regulamin2 (accessed on 15 August 2022) https://www.gov.pl/web/szczepimysie/do-wygarnia-zdrowie-i-miliony-zlotych-ruszaja-konkursy-dla-gmin (accessed on 15 August 2022)
Spain	Introduction of COVID-19 certificates in 10 regions and 2 cities. Required in bars, restaurants, nursing homes, health establishments as well as to access events.	22 November 2021	6 December 2021		https://elpais.com/sociedad/2021-11-26/certificado-covid-que-comunidades-han-aprobado-su-uso-y-para-que-actividades.html (accessed on 15 August 2022) https://www.abc.es/sociedad/abci-estas-son-comunidades-necesitar-pasaporte-covid-navidad-nsv-202112171646_noticia.html (accessed on 15 August 2022)
United Kingdom	Food, clothes, transportation and leisure discounts and vouchers for the vaccinated.	1 August 2021	1 August 2021		https://abcnews.go.com/Health/wireStory/pizza-shots-uk-targets-young-vaccine-incentives-79200767 (accessed on 15 August 2022) https://www.bbc.com/news/uk-58044088 (accessed on 15 August 2022)

**Table 2:** Change in number of daily administered doses before and after the policy interventions, that proved to be statistically significant.

Intervention	Type of Change	Time Period	Change in Number of Daily Administered Doses Total (95% CI)/Per Million Capita	Gain in Number of Doses Over a Time Period (Based on Counterfactual Analysis)
Announcement of cash incentive (“Freedom pass” in Greece)	Pulse	Immediate	12,354 (11,755–12,954)/1197	
Financial incentives for pharmacists and doctors (Greece)	Pulse	Immediate	1546 (1215–1878)/150	
Lottery and municipalities competition (Poland)	Pulse	Immediate	25,196 (15,023–35,371)/667	
Lottery (Russia)	Pulse	Immediate	9766 (4003–15,530)/67	
Introduction of COVID-19 certificates (Greece)	Step	Over 1 month	1500 (1284–1716)/145 immediately	830,956
Introduction of COVID-19 certificates (Russia)	Pulse and step	Over 1 month	−42,718 (−78,256–7180)/−293 and 43,775 (8201–79,349)/300 immediately	17,149,679
Introduction of COVID-19 certificates (Spain)	Pulse	Over 3 days	212,306/4544	
Introduction of COVID-19 certificates (Norway)	Gradual step	5 days	1308 (228–2388)/237 on day 4 −1399 (−2482–315)/−254 on day 5	
Introduction of employee vaccine mandate (Russia)	Pulse	Over 1,5 months	−43,429 (−78,063–8797)/−298 immediately	
Announcement of the universal vaccine mandate (Austria)	Step	Over 1 month	−3010 (−3592–2428)/−332 immediately	−1,696,266 (“missed” doses due to the policy change)
Introduction of the vaccine mandate for adults aged 60 and older (Greece)	Step	Immediate	−1932 (−2066–1798)/−187	
Introduction of vaccine mandate for adults 50 and older (Italy)	Pulse and step	Over 4 days	−43,358 (−53,057–33,659)/−720 on day 1 89,729 (76,849–102,610)/1489 on day 2 17,920 (8321–27,518)/297 on day 3 −76,621 (−86,530–66,712)/−1272 on day 4	

## Data Availability

Publicly available datasets were analyzed in this study. This data can be found here: [https://ourworldindata.org/coronavirus, accessed on 3 September 2022].
